# Fine‐scale genetic structure in rhizosphere microbial communities associated with *Chamaecrista fasciculata* (Fabaceae)

**DOI:** 10.1002/ece3.10570

**Published:** 2023-09-25

**Authors:** Mahboubeh Hosseinalizadeh Nobarinezhad, Lisa E. Wallace

**Affiliations:** ^1^ Department of Biological Sciences Mississippi State University Starkville Mississippi USA; ^2^ Department of Biological Sciences Old Dominion University Norfolk Virginia USA

**Keywords:** 16S rRNA, *Chamaecrista fasciculata*, fine‐scale genetic structure, ITS, rhizosphere microbial community

## Abstract

Soil microbiota of the rhizosphere are an important extension of the plant phenotype because they impact the health and fitness of host plants. The composition of these communities is expected to differ among host plants due to influence by host genotype. Given that many plant populations exhibit fine‐scale genetic structure (SGS), associated microbial communities may also exhibit SGS. In this study, we tested this hypothesis using *Chamaecrista fasciculata*, a legume species that has previously been determined to have significant SGS. We collected genetic data from prokaryotic and fungal rhizosphere communities in association with 70 plants in an area of ~400 square meters to investigate the presence of SGS in microbial communities. Bacteria of Acidobacteria, Protobacteria, and Bacteroidetes and fungi of Basidiomycota, Ascomycota, and Mortierellomycota were dominant members of the rhizosphere. Although microbial alpha diversity did not differ significantly among plants hosts, we detected significant compositional differences among the microbial communities as well as isolation by distance. The strongest factor associated with microbial distance was genetic distance of the other microbial community, followed by geographic distance, but there was not a significant association with plant genetic distance for either microbial community. This study further demonstrates the strong potential for spatial structuring of soil microbial communities at the smallest spatial scales and provides further insight into the complexity of factors that influence microbial composition in soils and in association with host plants.

## Graphical abstract

This study characterized fine‐scale patterns of microbial diversity in the rhizosphere of the host plant, *Chamaecrista fasciculata*. Diversity of bacteria and fungi at this small scale is correlated, exhibits an isolation by distance, and is best explained by environmental factors, rather than the host plant genotype.

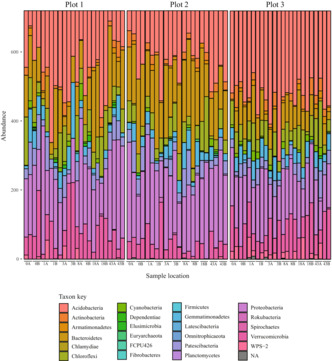

## 1 INTRODUCTION

According to Ettema and Wardle (2002), soil microorganisms, while nesting within large‐scale structures, regardless of topography and uniform soil texture tend to exhibit patchy distributions at the scale of centimeters to meters. They further believed that spatial patterns of soil biota at small scales, or fine‐scale genetic structure (SGS), are structured primarily by plant growth and spacing among them. Indeed, several studies suggest that plants are the most important factor shaping the rhizosphere microbiome, as evidenced by the fact that different plant species host specific microbial communities when grown in the same soil (e.g., Bazghaleh et al., 2015; Berendsen et al., 2012). Given that plant species differ in the quantity and quality of their belowground inputs to soil (Wardle et al., 2004), the structure of their rhizosphere microbial communities can be distinctive in composition and diversity (e.g., Costa et al., 2006; Garbeva et al., 2008). Rhizodeposits released by plants vary according to the age and development of plants (Inceoğlu et al., 2010; Philippot et al., 2013; Wagner et al., 2016), among species (Hacquard, 2016; Lemanceau et al., 2017) and even among different genotypes of the same species (Bazghaleh et al., 2015; Micallef et al., 2009; Wagner et al., 2016). It is also shown that microbial community composition can be significantly correlated with phylogenetic distance of host plants (Bouffaud et al., 2012). Thus, genetically similar host plants may have similar soil microbial communities, leading to potential genotype × genotype interactions in these situations. Because bacterial and fungal communities are frequently dispersal‐limited (Peay et al., 2012) and host plants influence microbial diversity and abundance, spatial autocorrelation of microbial communities in the rhizosphere is expected (Ettema & Wardle, 2002; Fierer & Ladau, 2012).

Few studies have investigated intraspecific plant genotypic influences on rhizosphere microbial communities, and most of these have only studied subsets of these communities, such as plant growth‐promoting bacteria (e.g., Hartman et al., 2017), rather than the whole microbiome. Thus, relatively little is known about the relationship between intraspecific genotypic variation of host plants and associated microbial communities outside model systems and under natural conditions (Schweitzer et al., 2011). Rhizobia have been extensively researched in numerous studies, but we do not know as much about other bacterial and fungal species present in the vicinity of roots. By investigating the genetic impact of a leguminous species on whole microbial communities in the rhizosphere and not just focusing on rhizobia, we can develop a better understanding of plant microbe interactions, particularly for the smallest of spatial scales.

*Chamaecrista fasciculata* (Michx.) Greene (Partridge Pea) is an annual, sub‐erect native legume that is widely distributed in the eastern U.S. Given its frequent occurrence in early successional habitats, it has great potential to influence below‐ground community composition. *C. fasciculata* is symbiotic with rhizobia (Parker & Kennedy, 2006) and numerous studies have documented diversity in *Bradyrhizobium* nodule symbionts (Dorman & Wallace, 2019; Nobarinezhad & Wallace, 2020; Parker, 2012, 2015; Parker et al., 2002; Parker & Rousteau, 2014).

In a previous study, we identified fine‐scale spatial genetic structure (SGS) for both host plants and nodulating rhizobia and found that plant genetic identity and geographic distance are significant predictors of the genetic structure of nodulating rhizobia of *C. fasciculata* (Nobarinezhad & Wallace, 2020). In the current study, we broaden our assessment of the same host plants used by Nobarinezhad and Wallace (2020) to determine if similar genetic structure exists in the bacterial and fungal communities of the rhizosphere of *C. fasciculata*. We tested the following hypotheses: (1) Rhizosphere microbial communities, encompassing bacteria and fungi, have varying composition at a local spatial scale, (2) This compositional variation exhibits genetic structuring that may be explained by (a) genetic structuring in host plants and/or (b) isolation by distance.

## 2 MATERIALS AND METHODS

### 2.1 Sampling and data collection

**FIGURE 1:**
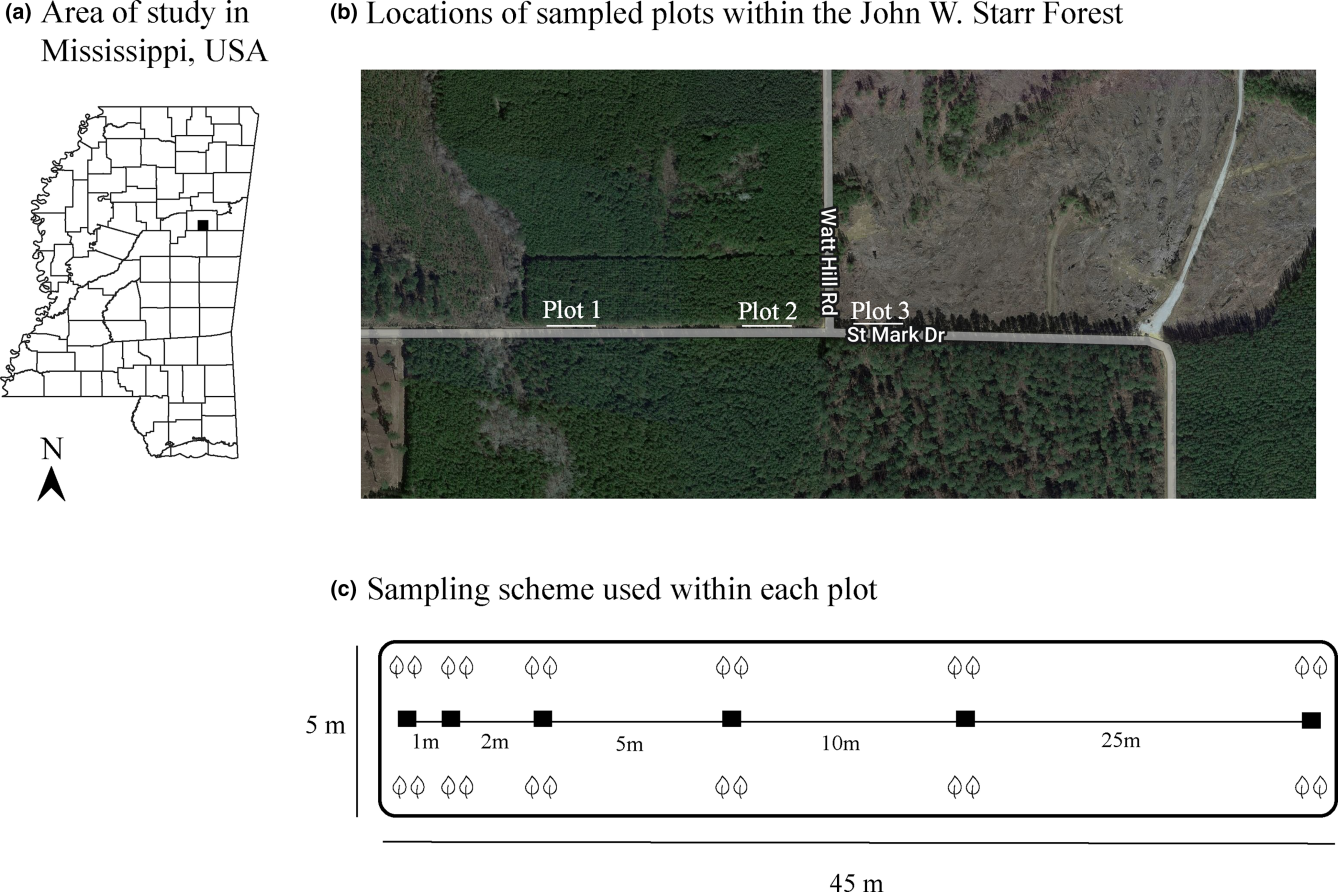
Study area in Mississippi (a), proximity of the three sampled plots (b), and sampling scheme used in each plot (c). At each location (boxes) in (c), two plants each were sampled (represented by the leaves) in opposite directions 0.5 m from a central transect, for a total of 24 plants sampled per plot. Sampling intervals from the start of the plot were 0 m and 1, 2, 5, 10, or 25 m from the previous point.

The study site was located within the John W. Starr Wildlife Management Area in Mississippi, USA at the edge of a *Pinus taeda* forest (Figure 1). To achieve uniform sampling distances, we established three rectangular plots, each ca. 45 m in length. The width of the plots was ca. 5 m as they were bordered by pine forest to the north and a gravel road to the south. The plants colonized this area naturally from seed. Within each plot, we sampled plants at distance intervals of 0, 1, 2, 5, 10, and 25 m from the previous point. At each distance interval, four plants were sampled, 0.5 m from a central line established in the plot, for a total of 24 plants per plot (Figure 1). In plot 2 at distance interval 10, we only sampled two plants due to the absence of four plants within 0.5 m of the central line. In total, 70 plants were sampled throughout this population, and sampling encompassed the entire area in which we observed the species growing. Whole plants, including roots, were carefully excavated from the soil. Each plant and its roots were stored individually in a plastic bag on ice in the field. Plants were kept at 4°C until processed within 24 h. Rhizosphere soil was removed from each plant by shaking the roots. Each soil sample was stored in a collection tube at −80°C until DNA extraction.

To acquire a better understanding of the potential variation in soil pH at a local scale, which has been identified as an important factor influencing soil bacterial communities (Fierer & Jackson, 2006), we combined samples of rhizosphere soil from the first three distance intervals (i.e., 0, 1, and 2) and from the last three distance intervals (i.e., 5, 10, and 25) for each plot. Soil then was pulverized, dried, and sent to Waypoint Analytical soil laboratory (Richmond, Virginia, USA) for pH analysis. Combining of soils was necessary because of the small amount of soil available in the rhizosphere and the desire to have estimates of soil pH from areas with actively growing plants. Additionally, as our study area encompassed a small spatial scale, unlike other experiments that studied larger scales (Fierer & Jackson, 2006), we did not expect to observe significant differences in soil pH levels among distance intervals.

For microbial community sequencing of rhizosphere soil samples, whole‐community DNA was extracted using the FastDNA™ SPIN Kit for soil isolation (MP Biomedicals). The concentration of the extracted DNA was determined using a Qubit 3 Fluorometer (ThermoFisher Scientific). By applying 319F and 806R primer pair sequences designed for the V3 and V4 region of the 16S rRNA gene (Holm et al., 2019) for eubacterial and archaeal microbes, and ITSF1/ITS2 for the fungal ITS1 gene (Sun et al., 2018), individual amplicons were produced using a two‐step PCR method as described in Holm et al. (2019). In the first PCR of 16S rRNA, each 12‐μm volume reaction contained 1× Phusion Taq master mix (New England Biolabs), step 1 forward and reverse primers, 319F and 806R, (0.4 μM each), 3% DMSO, and 5 ng genomic DNA. The thermal cycler program included an initial denaturation at 94°C for 3 min, 20 cycles of denaturation at 94°C for 30 s, annealing at 58°C for 30 s, elongation at 72°C for 1 min, and a final elongation step at 72°C for 7 min. For the first PCR of ITS1, each 12‐microliter volume reaction contained 10× Standard Taq (Mg‐free) reaction buffer (New England Biolabs), dNTP's (2 mM), MgCl2 (2 mM), Taq polymerase (0.2 units), 3% DMSO, forward and reverse primers, ITSF1/ITS2 (each 0.5 or 0.8 μM; the required concentration varied among DNA samples), and 10 ng of genomic DNA. The thermal cycler program for ITS1 included an initial denaturation at 94°C for 2 min, followed by 50°C for 1 min, and 72°C for 1 min; 30 cycles of denaturation at 94°C for 1 min, annealing at 50°C for 1 min, elongation at 72°C for 45 s, and a final elongation step at 72°C for 5 min. The successful amplification of PCR products was verified by running a sample using agarose gel electrophoresis. For both gene regions, the resultant amplicons were diluted 1:20, and 1 μL was used in the second PCR. The second amplification introduced an 8‐bp dual‐index barcode (Holm et al., 2019) to the amplicons, as well as the flow‐cell linker adaptors, using primers containing a sequence that anneals to the Illumina sequencing primer sequence introduced in step 1. The second PCR, library quantification, and Illumina sequencing were conducted at the Microbiome Service Lab at the University of Maryland School of Medicine following the methods in Holm et al. (2019). The 16S rRNA and ITS1 libraries of pooled amplicons were sequenced separately using 2 × 250 bp paired ends on a MiSeq instrument (Illumina).

### 2.2 Sequence data processing and data analyses

Following Berlanas et al. (2019), bioinformatics analysis and annotation of the data were carried out using QIIME2 software (Bolyen et al., 2019). Sequences were demultiplexed by their barcode at the University of Maryland and provided to us for the subsequent analyses described below. The primer sequences were removed from each read using q2‐cutadapt plugin, and sequence quality control and feature table construction were conducted using DADA2 pipeline (Callahan et al., 2016). Chimeras for combined runs were removed per the DADA2 protocol. Amplicon sequence variants (ASVs) generated by DADA2 were taxonomically classified using the scikit‐learn classifier (Pedregosa et al., 2011) trained with the SILVA v132 16S rRNA gene sequence database (Quast et al., 2012). For the ITS1 dataset, we processed sequence output as for the 16S rRNA data, with the addition of using q2‐itsxpress plugin to extract only the fungal ITS1 regions of the reads and using the UNITE reference database (UNITE Community, 2019) for taxonomic assignment. A total of nine samples (three in plot 1 and six in plot 3) did not return adequate sequence data and were removed from further analyses of fungal diversity. The output from QIIME2, including OTU and taxonomy tables, reference sequences, and the phylogenetic tree, was used as input for the subsequent analyses of alpha and beta diversity, which measures local diversity and turnover between samples, respectively. Alpha diversity was calculated for each of the 12 sample locations in each plot (i.e., sequences from the two plants sampled in the same direction from the central point at a given distance were pooled), whereas beta diversity was calculated between each pair of distance intervals (i.e., 1, 2, 5, 10, and 25 m) in each plot. These analyses were conducted separately for the bacterial and fungal isolates.

Biodiversity indices and ordination analyses based on taxonomic profiles were conducted in RStudio statistical software v. 1.1.456 (RStudio Team, 2022) using the vegan (Oksanen et al., 2018) and Phyloseq packages (McMurdie & Holmes, 2013). For each rhizosphere sample, sequences were rarified to simulate a similar number of reads per sample. A taxonomic bar plot was generated using rarified samples for each data set to better display the microbial structure. Alpha diversity, or the diversity of microbes within rhizospheres of plants at each sample location, can be described by richness, which is the number of distinct OTU's per sample, or evenness, which takes into account not only distinct OTU's but also their abundance within a sample. To calculate evenness, we used the Shannon Index. The Shannon Index and OTU richness were calculated for each sample using the Phyloseq package (McMurdie & Holmes, 2013). Kruskal–Wallis tests in the Qiime2 software (Bolyen et al., 2019) were used to determine if alpha diversity differed between pairs of rhizosphere samples. Shannon and evenness vectors output from QIIME2 were used as an input to measure differences between pairs of samples.

To evaluate beta diversity, which quantifies dissimilarity in rhizospheric soil composition between sample points, weighted UniFrac distance matrices (Lozupone & Knight, 2005) for each bacterial and fungal community were subjected separately to PERMANOVA (Anderson, 2001) using the adonis function in the vegan package (Oksanen et al., 2018) with 999 permutations to test for significance. Dissimilarity of communities associated with different sample points was visualized using principal coordinates analysis (PCoA).

We tested for fine‐scale genetic structure of the bacterial and fungal rhizosphere communities using Mantel correlograms between weighted UniFrac distance matrices (Lozupone et al., 2007; Pepe‐Ranney et al., 2016) of the bacterial or fungal sequences and spatial distance between sampled plants calculated using a modification of the haversine formula (Sinnott, 1984) in GenAlEx (Peakall & Smouse, 2012). Distance classes were set with upper limits of 1, 2, 3, 5, 7, 8, 10, 15, 17, 18, 25, 35, 40, 42, 43, 100, 200, 300, and 400 such that we could evaluate genetic structure within each plot as compared to larger distances between the plots. The maximum pairwise distance between plants within each plot was 43. Mantel correlograms were performed using PASSaGE v.2 (Rosenberg & Anderson, 2011) with 9999 permutations to test for significance of Mantel *r* for each distance class.

Multiple matrix regression (MMR) was used to assess the association between genetic distance of microbial communities based on weighted UniFrac distance matrices (Lozupone et al., 2007; Pepe‐Ranney et al., 2016) and three predictors: geographic distance, host plant genetic distance, and other microbial community genetic distance. Plant hosts were genotyped at 14 microsatellite loci, and these data were used to calculate pairwise genetic distances for all sampled plants using GenAlEx (Peakall & Smouse, 2012), as detailed in Nobarinezhad and Wallace (2020). Geographic distances between sampled host plants were calculated from coordinates of the sampled plants as described above. Univariate and multivariate MMR analyses were conducted using the MMRR script of Wang (2013) in R (RStudio Team, 2022) with the explanatory matrixes of geographic distance, plant host genetic distance, and genetic distance of the rhizosphere microbial community (i.e., fungi for the bacterial dataset and bacteria for the fungal dataset) and the response matrix of genetic distances of bacterial or fungal communities, respectively. Significance of each model was tested using 9999 permutations.

## 3 RESULTS

**FIGURE 2:**
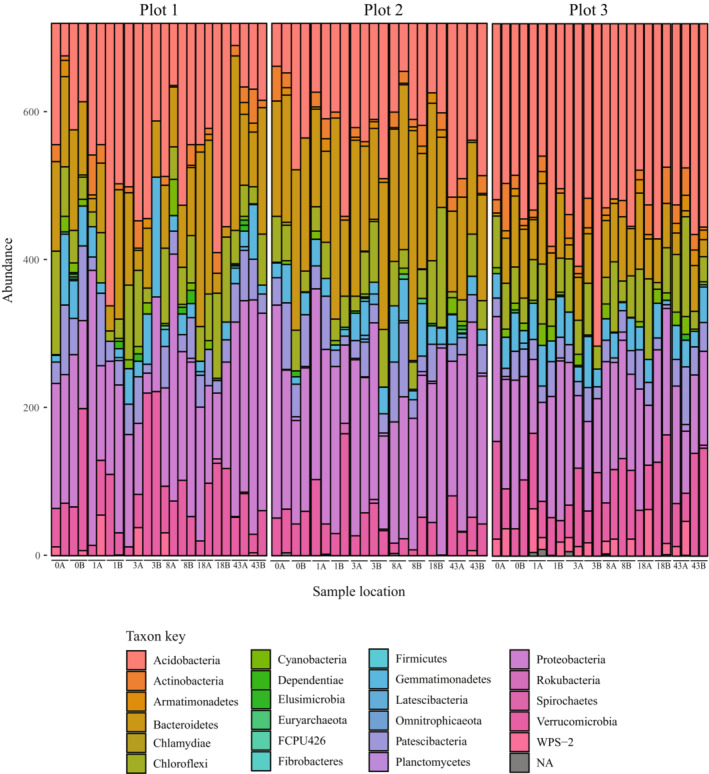
Taxonomic classification of microbial reads in rhizospheric soil at the phylum level for bacterial communities of each host plant. The site of each sample is indicated along the *x*‐axis.

**FIGURE 3:**
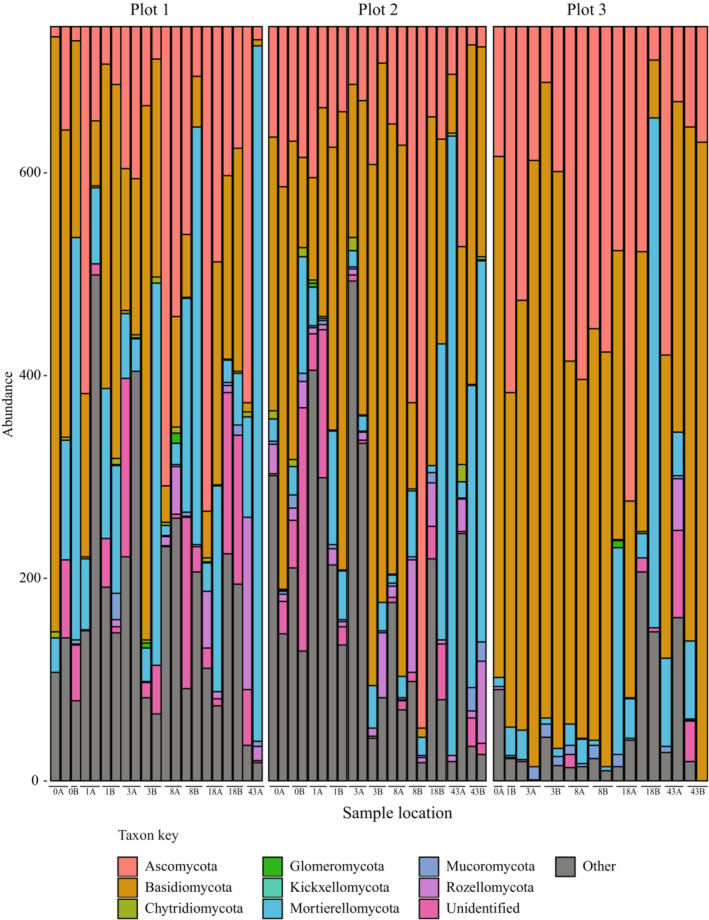
Taxonomic classification of microbial reads in rhizospheric soil at the phylum level for fungal communities of each host plant. The site of each sample is indicated along the *x*‐axis.

**FIGURE 4:**
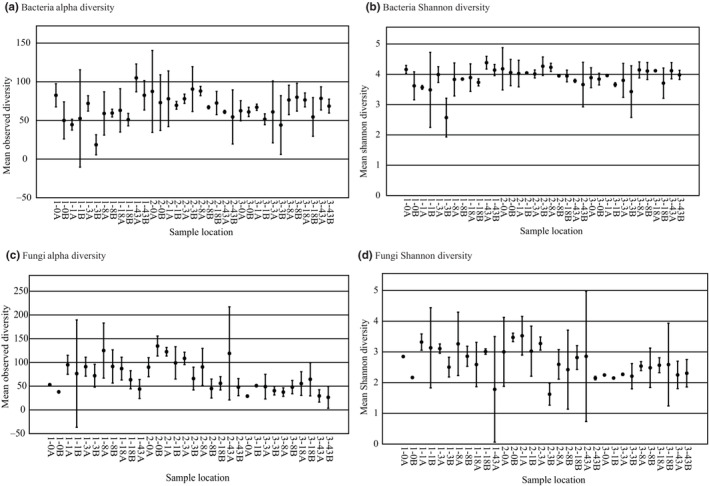
Boxplots of the number of observed OTUs and the Shannon diversity index for bacterial (a, b) and fungal (c, d) communities sampled from rhizosphere samples collected at six specific distances in each plot. The site of each sample is indicated along the *x*‐axis.

**FIGURE 5:**
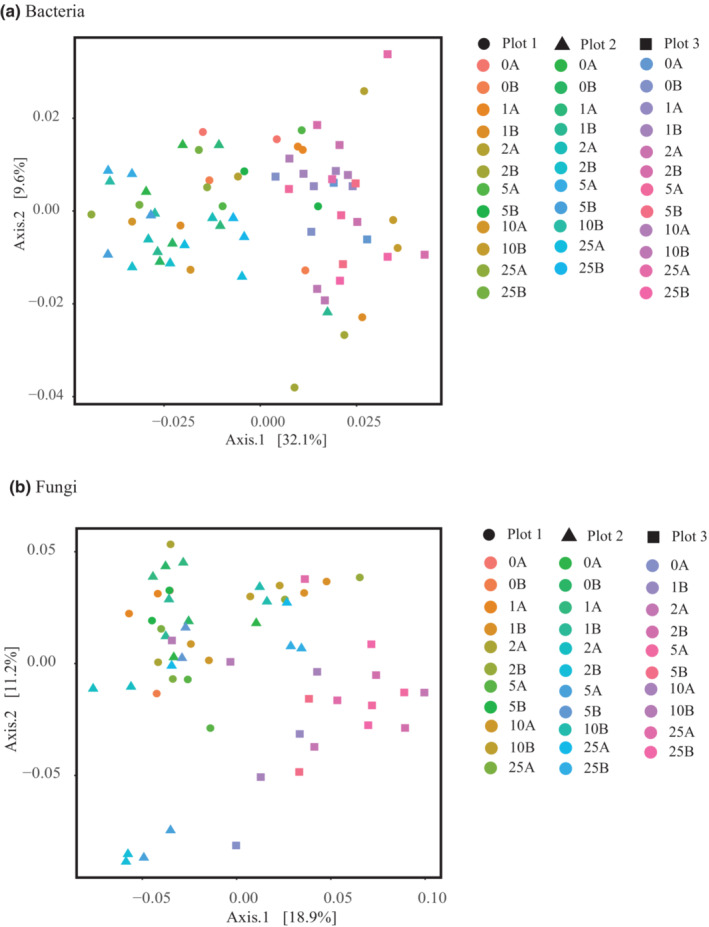
Plot of the first two components from a principal coordinate analysis showing beta diversity among rhizosphere communities in each plot. The first number in each sample name indicates the plot number and the second number represents the specific distance at which the sample was collected for (a) bacterial communities and (b) fungal communities. The first two axes account for approximately 41.7% of the total variance (*p* = .001) for bacterial communities and approximately 30.1% of the total variance (*p* = .001) for fungal communities.

The pH for rhizospheric soil among the first three sample locations and the last three sample locations, respectively, was 5.9 and 6.7 in plot 1, 6.5 and 5.4 in plot 2, and 5.3 for both locations in plot 3. Bacteria of Acidobacteria, Protobacteria, and Bacteroidetes (Figure 2) and fungi of Basidiomycota, Ascomycota, and Mortierellomycota (Figure 3) were dominant in the rhizosphere soils for all plots. No significant differences in alpha diversity of bacterial communities nor fungal communities were detected among plants in any of the plots nor among the three plots (Table 1, Figure 4). However, beta diversity did vary among samples (Table 1, Figure 5), and these values were significantly different from one another for both bacterial (PERMANOVA: pseudo‐*F* = 2.08, *p* = .001) and fungal data sets (PERMANOVA: pseudo‐*F* = 2.71, *p* = .001). In PCoA plots, the first two components accounted for approximately 42% of the total variance observed among bacterial communities and 30% of the total variance among fungal communities sampled among different plant hosts. Bacterial sequences from plot 1 are scattered in the PCoA plot while samples from plots 2 and 3 appear more distinct from one another (Figure 5a). In the fungal PCoA plot, sequences retrieved from plots 1 and 2 overlapped to some extent but these are distinct from those in plot 3 (Figure 5b).

**TABLE 1:** Shannon and evenness indices for bacterial and fungal communities within and between the three sampled plots.

Microbial community	Index	Plots	*H* ^a^	*p*‐Value
Bacterial	Shannon	Within	45.62	.08
		Among	2.68	.26
	Evenness	Within	39.89	.22
		Among	0.49	.78
Fungal	Shannon	Within	37.36	.19
		Among	8.98	.11
	Evenness	Within	32.99	.36
		Among	0.87	.64

*Note*: *p*‐Values were determined using Kruskal–Wallis tests.

^a^ *H* = the variance of the ranks among groups.

**FIGURE 6:**
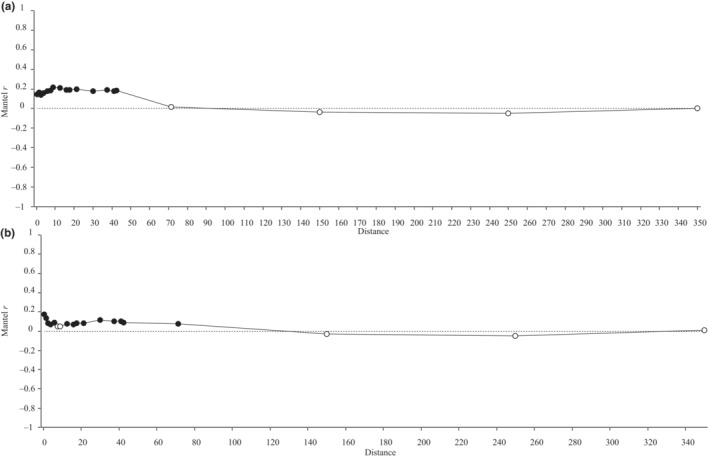
Mantel correlogram plots depicting the relationship between geographic distance and genetic distance of bacterial (a) or fungal (b) rhizosphere communities across varying distance classes. Mantel *r* values that are significant (*p* < .05) across 9999 permutations are indicated by black circles; non‐significant values are in white. Distance classes between 0 and 43 represent plants sampled within a plot and those >43 are between plots.

**FIGURE 7:**
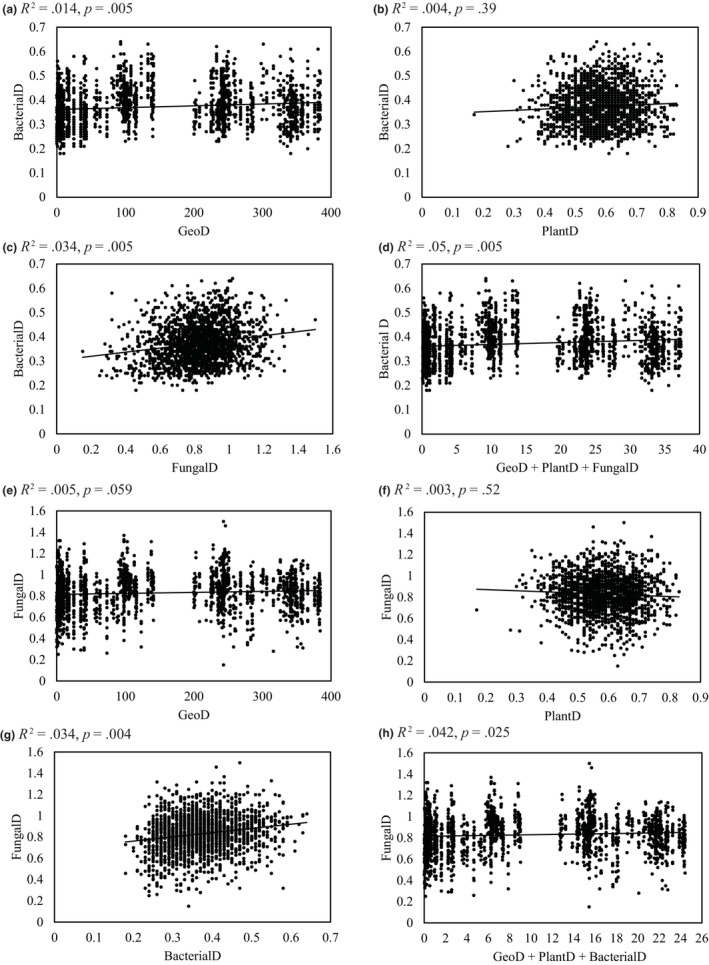
Scatterplots depicting the relationship between geographic distance, host plant genetic distance, and other microbial community distance on bacterial (a–d) and fungal (e–h) communities observed across the three plots based on multiple matrix regressions (MMR). Matrix regressions were conducted for genetic distance of bacterial (BacterialD) and (a) geographic distance (GeoD), (b) host plant genetic distance (PlantD), (c) fungal distance (FungalD), and (d) the multivariate model of GeoD + PlantD + FungalD. Matrix regressions were conducted for genetic distance of fungal (FungalD) and (e) geographic distance (GeoD), (f) host plant genetic distance (PlantD), (g) bacterial distance (BacterialD) and (h) the multivariate model of GeoD + PlantD + BacterialD.

We found significant fine‐scale genetic structure within plots for both bacterial and fungal communities, but no significant structure was detected at distances >43, which correspond to between‐plot differences only (Figure 6). Mantel *r* values at distances of 0–43 ranged from 0.14 to 0.22 for bacteria and 0.05–0.17 for fungi. Multiple matrix regressions for each of the microbial communities did not indicate a significant effect of plant genetic distance on genetic structure of bacterial nor fungal communities (Table 2, Figure 7). Geographic distance was significantly associated with genetic distances of bacteria in univariate and multivariate analyses, but for fungal genetic distance geographic structure was only marginally significant in the univariate analysis. Genetic distance of fungi and bacteria had the highest coefficient for the bacterial and fungal genetic distances, respectively, in multivariate analyses, and these factors were significant (*p* < .05) in both analyses. Although significant (*p* < .05) predictors were identified, still relatively little of the observed genetic distance variation was explained by them (Table 2).

**TABLE 2:** Results from multiple matrix regression considering multivariate and univariate analyses with predictors plant genetic distance, geographic distance, and either bacterial or fungal genetic distance.

	Univariate		Multivariate	
	*R* ^2^	*p*	Coefficient	*p*
Bacteria				
Plant genetic distance	.004	.39	.054	.449
Geographic distance	.014	.005	.097	.012
Fungal genetic distance	.034	.005	.180	.003
Full model			*R* ^2^ = .05, *p* = .005	
Fungi				
Plant genetic distance	.003	.52	−.077	.349
Geographic distance	.005	.059	.064	.116
Bacterial genetic distance	.034	.004	.181	.005
Full model			*R* ^2^ = .042, *p* = .025	

*Note*: The response variable in each analysis was bacterial or fungal genetic distance.

## 4 DISCUSSION

The rhizospheric communities identified for *C. fasciculata* appear to be common to plants in natural ecosystems, suggesting a role for phylogenetic conservatism in soil microbes of terrestrial plants. We found that Acidobacteria, Protobacteria, and Bacteroidetes were the dominant bacteria and Basidiomycota, Ascomycota, and Mortierellomycota were the dominant fungi in the *C. fasciculata* rhizosphere. These microbial groups play a variety of roles in supporting ecosystem functioning (Table 3) and are consistent with previous reports of major constituents of legume rhizosphere microbiomes (Hartman et al., 2017; Mendes et al., 2014; Sugiyama et al., 2014; Xiao et al., 2017). Given the presence of SGS in *C. fasciculata* (Nobarinezhad & Wallace, 2020), we hypothesized that rhizosphere microbial composition would also vary at this scale. While we found support for SGS in both bacterial and fungal communities, surprisingly, we did not find that host genotype per se is significantly associated with turnover in rhizosphere communities. Rather, significant predictors of the microbial SGS include geographic distance, which may result from dispersal limitation, and the reciprocal microbial community, which adds to growing evidence of the presence and importance of tripartite interactions as important to plant function and community structure (Afkhami et al., 2020).

**TABLE 3:** Primary microbial phyla identified in the rhizosphere of *Chamaecrista fasciculata* and their roles in ecosystem functioning.

Phylum	Functions	References
Bacteria		
Acidobacteria	Nitrogen assimilation, carbon usage, metabolism of iron, antimicrobials, and transporters	Parsley et al. (2011), Faoro et al. (2012), Navarrete et al. (2013) and Mendes et al. (2014)
Proteobacteria	Carbon, sulfur, and nitrogen cycling	Manz et al. (1992), Moulin et al. (2001) and Spain et al. (2009)
Bacteroidetes	Degradation of complex carbohydrates, including polysaccharides, enabling soluble sugars to be made available to other organisms; recycling of carbon, nitrogen, and water	Thomas et al. (2011) and Griffiths and Gupta (2001)
Fungi		
Basidiomycota	Primary decomposers of recalcitrant components of plant litter; at least 4500 species are ectomycorrhizae in the roots of vascular plants	Rayner and Boddy (1988) and Watling (1995)
Ascomycota	Carbon and nitrogen cycling in arid ecosystems, soil stability, plant biomass decomposition, and endophytic interactions with plants	Crenshaw et al. (2008), Green et al. (2008) and Challacombe et al. (2019)
Mortierellomycota	Saprotrophs	Zhang et al. (2019)

Even though alpha diversity was not significantly different across host plants for the bacterial nor fungi datasets (Table 1), each host plant exhibited a unique microbial signature (Figures 2 and 3), suggesting that *C. fasciculata* plants at this location are equally likely to harbor diverse rhizosphere communities. Variation in total species diversity (Figure 4), as well as differences in the compositional makeup of rhizosphere communities, particularly for fungi (Figure 2b), indicates heterogeneity, supporting our hypothesis that rhizosphere composition is locally variable. Other studies have noted mixed results on the association between host plant diversity and overall diversity of microbes in their rhizospheres. For example, Zverev et al. (2021) did not find a correlation between alpha diversity of plants and rhizosphere bacteria in farmed and fallow fields, but Singh et al. (2022) reported that alpha diversity varied among rice cultivars. These studies suggest a complex interplay of factors likely determine the level of microbial diversity associated with plants, as well as the specific types of microbes present.

A comparison of alpha diversity revealed relatively higher bacterial than fungal diversity as the mean number of OTU's for bacterial and fungal communities was 72 and 66.88, the mean Shannon index was 3.89 and 2.67, and the mean evenness was 0.94 and 0.64, respectively. Except for experimental studies on microbial diversity in stressful environments, such as drought, disease, and contaminated or nutrient‐deficient soil, few studies have compared bacterial and fungal abundancy in rhizospheric soil of a wild legume. In an agricultural system, Essel et al. (2019) found similar abundance of the dominant bacteria, including Actinobacteria, Proteobacteria, Chloroflexi, Acidobacteria, and *Planctomycetes*, and dominant fungi, including Ascomycota and Basidiomycota, among tillage treatments in a crop rotation of wheat (*Triticum aestivum* L.) and pea (*Pisum arvense* L.) Higher bacterial diversity in our study might be explained by higher tendency for symbiotic relationships between legumes and bacteria for nitrogen fixation and assimilation, carbon and sulfur cycling, metabolism of iron, antimicrobial and transporter properties (Manz et al., 1992; Mendes et al., 2014; Moulin et al., 2001; Navarrete et al., 2013; Parsley et al., 2011). Additionally, because mycorrhizal fungi are often supported by bacteria (Basiru et al., 2023; Emmett et al., 2021), this could result in higher bacterial diversity among our samples.

The reasons for microbial turnover leading to significant SGS include abiotic factors, dispersal limitation, and host plant diversity, among others. Soil pH has been recognized as important in determining soil microbial composition generally, but we suggest it is unlikely to be a strong determinant at small scales except in areas of edaphic or vegetation transitions. Fierer and Jackson (2006) found that the diversity of microbiomes in soils with pH 5.3–7.4, which is the range pH that we measured for our studied plots, is not highly variable. Thus, we suggest that rhizosphere microbial community composition and structure at the scale of our study are not likely to have been influenced by soil pH.

We previously identified SGS of *C. fasciculata* at the study site (Nobarinezhad & Wallace, 2020), and in the current study we also found significant differences in beta diversity of bacterial and fungal communities that is consistent with isolation by distance at spatial scales up to 25 m or within plots. Significant genetic structure was not detectable at larger distances or between plots. Other studies indicate that soil microbial communities are frequently spatially structured in wild and agricultural settings, but the distance at which this structure develops can vary across plant hosts and habitats. For example, Horner‐Devine et al. (2004) found that over distances of centimeters to hundreds of meters in salt marsh sediments, bacterial communities located close together were more similar in composition than communities located farther apart. In a study of *Pseudomonas* strains, a negative correlation was found between the genetic similarity of isolates and geographic distances ranging from 5 m to 80 km (Cho & Tiedje, 2000). Franklin and Mills (2003) documented microbial distance–decay patterns at smaller scales (2.5 cm to 11 m) and reported a significant distance–decay relationship with a scale‐dependent slope.

Numerous studies have experimentally determined that host genotype influences microbial composition, including in soybeans (Liu et al., 2019), potatoes (Weinert et al., 2011), and maize (Emmett et al., 2017). Variation in rhizosphere composition across soybean genotypes might be influenced by the specific profile of flavonoids and isoflavonoids, essential components involved in signaling between legumes and symbiotic rhizobia during nodule formation (White et al., 2015). For a wild species, *Spartina alterniflora* Loisel., significant differences in bacterial community composition and diversity between bulk and rhizosphere soil was also dependent on genetic variation of host plants (Zogg et al., 2018). Interestingly, our MMR results do not support host plant variation as a primary predictor of genetic structure in their rhizosphere communities. While the microsatellite loci we used are well‐suited for fingerprinting individual plants, they may not reflect functional aspects that influence microbial recruitment in the rhizosphere.

MMR analyses for bacterial communities revealed significant influence of geographic distance and fungal genetic distance on community structure and bacterial genetic distance alone for structure of fungal communities. Since genetic distances of both bacteria and fungi were significantly associated with geographic distance in univariate analyses, this result could reflect the similar response of these communities to spatial factors in our study. But reciprocal effects of bacteria and fungi on each other have been noted by other authors (Agler et al., 2016; Basiru et al., 2023; Emmett et al., 2021). For example, arbuscular mycorrhizae harbor their own microbiome (Basiru et al., 2023; Emmett et al., 2021), which could influence beneficial and harmful interactions between colonized plants and other microbes in the rhizosphere. Agler et al. (2016) identified microbial “hubs” or taxa of disproportionate influence on other microbes in the phyllosphere of *Arabidopsis thaliana* (L.) Heynh. and suggested that host plant genotype most strongly shapes the associated microbial community when it directly affects colonization of hub taxa. Thus, while it is commonly believed that host plant traits play a role in determining the composition of their rhizosphere communities, such effects may be diluted by microbe‐microbe interactions that take place after initial colonization by specific “hub” microbes. The presence of diverse symbioses common to many legumes, and discussed above, may also influence the structure of microbial communities of legumes in unique ways (e.g., Larimer et al., 2014). Such tripartite interactions, as well as the potential for them to be context‐dependent (Ossler et al., 2015), makes teasing out the individual influence of host plant genotype and other factors difficult.

The amount of variation in microbial distances explained by the tested variables is rather low, suggesting that other factors that we did not test are also likely important. Plant associations with soil microorganisms are context‐dependent, thus their effects on soil microbiota are often difficult to predict (Fierer, 2017). Environmental conditions can vary considerably across the distinct microbial habitats found in soil, including the rhizosphere, preferential water flow paths, and animal burrows. For example, oxygen concentrations can vary from 20% to <1% from the outside to the inside of soil aggregates that are only a few millimeters in size and as a result influence bacterial content and activity of that soil aggregate (Wilpiszeski et al., 2019). There have been several studies on viruses and their role in the soil microbiome, and phages that target specific bacteria, including *Rhizobium* spp. (Frampton et al., 2012; Kimura et al., 2008), but the overall effects of viruses on the composition and activity of the soil microbiome remain poorly understood. Studies that explore more directly traits and genes involved in symbiotic interactions with soil microbiota may reveal a stronger role for the plant host to influence the composition of its rhizosphere for *C. fasciculata*. Additionally, evaluation of temporal changes in microbial community structure over the lifespan of *C. fasciculata* would provide a deeper understanding of how the rhizosphere changes and the factors influencing those changes.

## 5 CONCLUSIONS

Our results contribute to a common finding that soil microbial communities are spatially structured. Thus far, the studies that have investigated these effects have primarily focused on specific rhizobacterial communities, such as plant growth‐promoting bacteria, and primarily on model legumes in agricultural settings and controlled environments. We studied a non‐model native legume in natural setting and showed that plants in physical proximity show significantly higher relatedness than those more distant from one another. Our data suggest that physical distance is important in shaping the genetic structure and diversity of bacterial and fungal communities of the rhizosphere and that the diversity of these communities may be influenced by each other. Our results, which are consistent with other fine‐scale microbial studies suggest that large‐scale studies of microbial diversity might have under‐sampled microbial diversity, which could result in a biased view of the spatial scaling of microbial biodiversity and the incorrect conclusion that the spatial scaling of microbial biodiversity is different from that of plant diversity. Given that under‐sampling could result in the observation of specifically flat or nonexistent rates of distance–decay, it increases the importance of sampling effort in describing diversity patterns.

## AUTHOR CONTRIBUTIONS

**Mahboubeh Hosseinalizadeh Nobarinezhad:** Conceptualization (equal); data curation (equal); formal analysis (lead); funding acquisition (equal); investigation (lead); methodology (equal); writing – original draft (lead); writing – review and editing (equal). **Lisa E. Wallace:** Conceptualization (equal); data curation (equal); formal analysis (supporting); funding acquisition (equal); investigation (equal); methodology (equal); project administration (lead); resources (lead); supervision (lead); writing – review and editing (equal).

## FUNDING INFORMATION

This research was funded by the Biology Faculty Fund of Mississippi State University and the J. Robert Stiffler Endowment at Old Dominion University.

## CONFLICT OF INTEREST STATEMENT

The authors declare no conflict of interest.

## Data Availability

Microbial sequences are deposited in the NCBI Sequence Read Archive (SRA) under BioProject PRJNA978088 (16S sequences) and BioProject PRJNA978088 (ITS sequences).
